# Conservation demands safe gene drive

**DOI:** 10.1371/journal.pbio.2003850

**Published:** 2017-11-16

**Authors:** Kevin M. Esvelt, Neil J. Gemmell

**Affiliations:** 1 MIT Media Lab, Massachusetts Institute of Technology, Cambridge, Massachusetts, United States of America; 2 Department of Anatomy, University of Otago, Dunedin, New Zealand

## Abstract

Interest in developing gene drive systems to control invasive species is growing, with New Zealand reportedly considering the nascent technology as a way to locally eliminate the mammalian pests that threaten its unique flora and fauna. If gene drives successfully eradicated these invasive populations, many would rejoice, but what are the possible consequences? Here, we explore the risk of accidental spread posed by self-propagating gene drive technologies, highlight new gene drive designs that might achieve better outcomes, and explain why we need open and international discussions concerning a technology that could have global ramifications.

In 2016, New Zealand boldly announced a program to eliminate all its rats, possums, and stoats by 2050. These invasive species cause enormous damage to the New Zealand flora and fauna, with rats and possums also imposing a substantial economic burden [1].

Limiting the populations of these invasive predators has been a key conservation goal for decades. Thanks to years of awareness campaigns and extended discussions, there is a strong public sense that these species are not part of the "natural" fauna [2]. We can now control and even remove many of these species, creating small “pest-free” sanctuaries where many native species, including kiwis, now have a realistic chance for survival [1,3]. Larger-scale invasive species removal is possible with current technologies; New Zealand has already eradicated invasive predators from 10% of its offshore island area [3], but it is time-consuming and expensive [1,4]. Predator Free 2050, a new company responsible for directing a significant amount of Crown investment into the Predator Free Programme [5], is now exploring technological solutions through which eradication can be achieved quickly and cheaply at landscape scale as a key step towards preserving the nation’s biodiversity [6]. One promising new approach involves the use of “gene drive” systems that promote the inheritance of a particular genetic variant to increase its frequency in a population (Fig 1).

**Fig 1:**
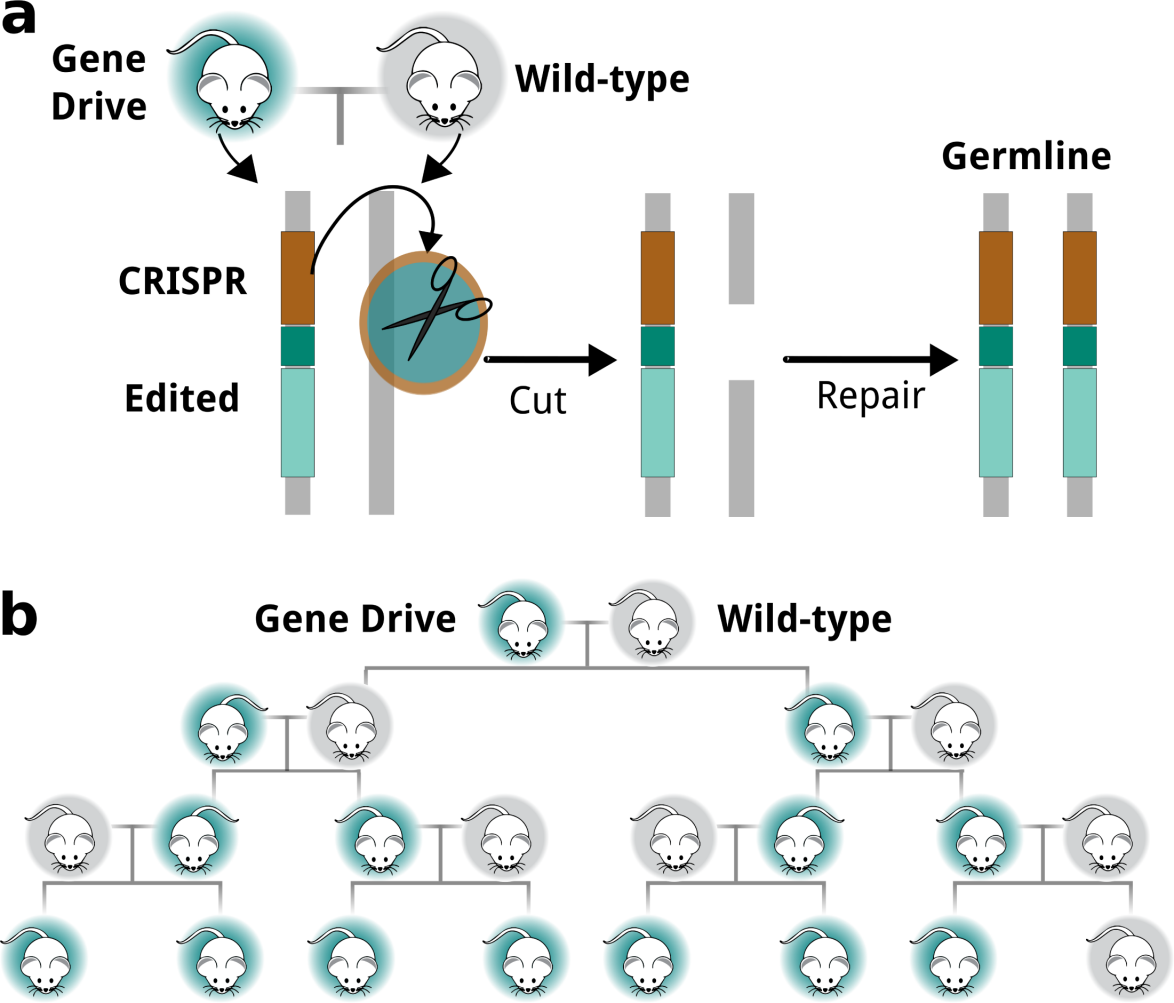
Gene drives distort normal patterns of inheritance. Normally, we receive 1 of 2 copies of a given gene from either parent, with a 50:50 chance of each copy being passed on. Gene drive systems distort that rule, promoting the inheritance of a particular copy of a gene from the parent to offspring. (a) CRISPR-based gene drive systems cut the equivalent allele on the wild-type chromosome, causing the cell to copy them via homology-directed repair. (b) Converting heterozygotes to homozygotes in the germline guarantees inheritance, enabling rapid spread through populations. This distortion in and of itself is relatively harmless, but when coupled to a genetic trait that affects an individual’s survival or ability to reproduce, it becomes a powerful tool that can be used for population control or even local elimination. CRISPR, Clustered Regularly Interspaced Short Palindromic Repeats.

There are many types of naturally occurring gene drive systems [7,8], but the advent of Clustered Regularly Interspaced Short Palindromic Repeats (CRISPR) genome editing has catapulted prior theoretical speculation into reality, at least in the laboratory [9,10]. Encode a desired genomic change along with the components of the CRISPR system, and it will cut and replace the original sequence with the new version in each generation. While recent reports have emphasized the inevitable rise of resistant mutations that block cutting by the nuclease [11–13], models from multiple groups [14–16] predict that using CRISPR to cut many sites within genes that are important for fitness [9] should reliably overcome this problem because any incorrect repair event that might otherwise generate a resistant allele will be very costly due to inactivating the target gene [9,14,15].

As first described by Austin Burt, nuclease-based gene drive systems can suppress or locally eliminate populations by disrupting recessive genes needed for fertility [17]. This type of drive system can spread because fertile organisms that inherit only 1 copy still pass it on to all offspring; those that inherit a copy from each parent will be born infertile, eventually causing the population to crash. Alternatively, the drive system can bias the sex ratio towards males. Either form of genetic population suppression would be extremely useful for conservation and, unlike traps and poisons, would not cause any animals to suffer. However, tackling cosmopolitan species such as rats with this strategy would likely have worldwide effects [9].

## Conservation and invasiveness do not mix

Numerous researchers and organizations have proposed the use of gene drive systems for conservation. Indeed, one of us (K.E.) included conservation as a potential application in the first publication describing standard CRISPR-based gene drives [9]. We now believe that inclusion was a mistake: such drive systems lack control mechanisms and are consequently highly invasive.

Suppose New Zealand were to release rats carrying a self-propagating CRISPR-based gene drive that targets a large number of conserved sequences within recessive fertility genes. According to recent modelling of island rodent populations subjected to different kinds of suppression gene drives, this approach would reliably eliminate the local invasive rodent population [16]. However, gene drive organisms would be present on the island for several years.

Because the introduction of only a handful of organisms may be sufficient for a drive system to invade a new population [18], any extended residence time provides an opportunity for the construct to hitch a ride to other islands and continents before it eliminates the local population and extinguishes itself [19]. Even if the gene drive rats did not manage to stow away on their own, past experience in the biocontrol sphere suggests there is a very high likelihood that they would be moved deliberately [20,21] to reduce economic costs that rats impose on many industries [22]. The calicivirus responsible for Rabbit Haemorrhagic Disease was smuggled into New Zealand to mitigate economic damage estimated to range from US$7–US$50 million per year [20,23]; in contrast, estimated annual losses to rats in the United States alone total US$19 billion [22]. With such strong economic incentives, it is safe to assume that some individuals will seek advantage through deliberate transport.

The bottom line is that making a standard, self-propagating CRISPR-based gene drive system is likely equivalent to creating a new, highly invasive species: both will likely spread to any ecosystem in which they are viable, possibly causing ecological change.

## Unsafe in any endemic environment

We are highly skeptical that it would be safe to release a self-propagating drive system capable of spreading beyond the target local population unless international spread is the explicit goal. Even building such a construct in laboratory containment within a region harboring the target species poses the risk that an accidental escape might eventually affect everyone who shares an ecosystem with that species. For rats and mice, that constitutes most people on Earth. While these species are pests in many contexts, they also underpin important ecosystem services in large areas of the globe (although there is little research that quantifies such effects) [24] and have cultural significance and value to many [25].

Set against this reality is the fact that individual nations, and perhaps especially New Zealand, could greatly benefit from releasing gene drive systems within their borders. However, moving forward without the permission of every other country harboring the target species would be highly irresponsible. Even assuming that national sovereignty is morally irrelevant, the social and diplomatic consequences of an unconstrained release should give us pause.

CRISPR-based gene drive is arguably the technology most likely to help eradicate human scourges such as malaria and schistosomiasis. It would be a profound tragedy if New Zealanders—or anyone else—inadvertently caused an international incident and consequent loss of public confidence in scientists and governance that interfered with its use. Going by past examples of accidents and misconduct in diverse fields of science, and particularly the effects of tragic outcomes in gene therapy trials, any unauthorized release of a gene drive system would quite likely delay applications by a decade or more [26]. For malaria alone, the cost of that delay could be measured in millions of otherwise preventable deaths [27].

To any who are still unfazed: Do we want a world in which countries and organizations routinely and unilaterally alter shared ecosystems regardless of the consequences to others? Isn’t that selfish and narrow focus the very philosophy that conservationists oppose?

## Reasons we might be wrong

Given this litany of reasons to be cautious, it’s fair to ask why anyone would consider building a standard CRISPR-based gene drive system for conservation. First, there is no obvious harm in developing the technology using appropriate laboratory safeguards [28]. Second, our understanding of the invasiveness problem is not widely shared: the 2016 US National Academies’ report on self-propagating gene drive explicitly recommended field trials, even though this would likely amount to uncontrollable release if gene drive systems are highly invasive. Third, we cannot be sure that the unauthorized release of a gene drive system would generate a social backlash; if suitably effective, it might even be applauded, thereby accelerating deployment of gene drive systems to solve different problems. Fourth, ecological consequences are uncertain; Eurasia might actually do quite well without black and brown rats—certainly food spoilage would lessen.

The problem is that each of these reasons requires us to be lucky. Accidental escape and deliberate unauthorized transport are not guaranteed, but they remain very real possibilities. As much as we may wish to discount the potential ecological complications of a suppression drive impacting the native ecosystems of invasive species, it remains a legitimate concern. The same applies to the risk of social or diplomatic backlash and the dangerous precedent set by deliberately using a broadly invasive tool to solve a local problem. History suggests that safety engineering becomes a primary concern only after a well-publicized disaster. That is not a pattern we care to perpetuate.

Now is the time to be bold in our caution. In our view, it is wise to assume that invasive and self-propagating gene drive systems are likely to spread to every population of the target species throughout the world. Accordingly, they should only be built to combat true plagues such as malaria, for which we have few adequate countermeasures and there is a realistic path towards an international agreement to deploy among all affected nations. If there is no such path—as is likely when the notion of a safe field trial is likely to be an oxymoron—then it is best if we refrain from developing self-propagating gene drive systems in the target species at all. For the handful of cases such as malaria for which a plausible path exists, the research should be undertaken in laboratories located far from existing populations of the target species and be performed in concert with extensive community engagement efforts in those nations likely to be affected. This is precisely the strategy being pursued by “Target Malaria” [29]. For all other candidate applications, including in New Zealand, we should not even consider building drive systems likely to spread indefinitely beyond the target area.

## Start small and scale up

Is there a way to salvage the potential benefits of self-propagating CRISPR-based drive systems for conservation? In theory, a “precision drive” system could specifically cut mutations that are fixed in an invasive population due to a founder effect yet rarely found elsewhere in the world [9,12]. At least 3 such mutations would need to be targeted per invasive population to prevent the rise of resistant alleles that block cutting [14–16] per drive system, and if the mutations are not all fixed, multiple such drive systems would likely be required. Crucially, each unique mutation must be located within an essential gene and must create a novel protospacer-adjacent motif (PAM) sequence that will permit it to be cut by a CRISPR nuclease. This is necessary because guide RNAs targeting any other type of mutation could evolve to permit spread in populations elsewhere. Whether sufficient unique mutations of this type already exist in different invasive populations remains to be determined.

**Fig 2:**
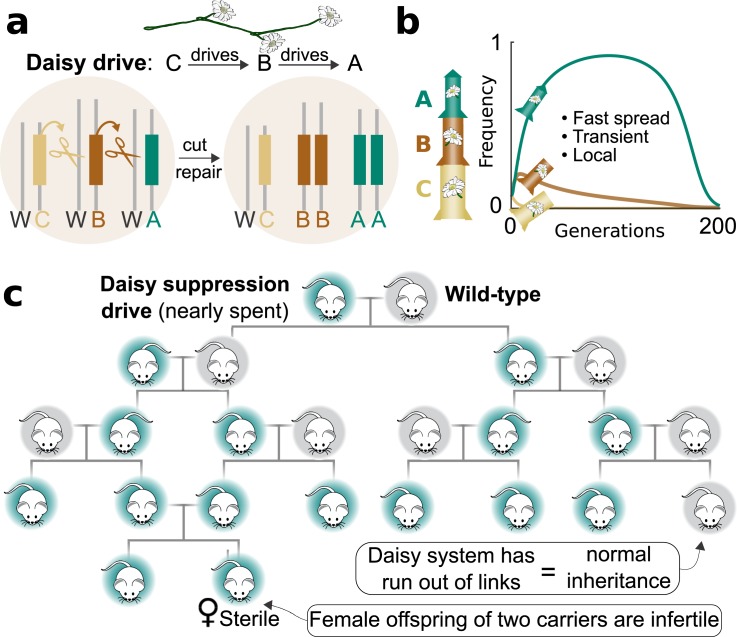
Daisy chain drive systems offer localized suppression. (a) In a daisy chain drive system, the CRISPR components are separated and arranged so that each daisy element drives the next in the chain. The element at the end, in this case C, is not copied and is lost in half of offspring. In these organisms, B is no longer copied and is lost in turn; this process continues until the drive system stops. (b) The loss of nondriving elements to natural selection is analogous to gravity on a rocket. Adding more elements to the daisy chain allows the system to spread further before it runs out of genetic fuel and halts. (c) Daisy drive systems can suppress local populations if the effector element disrupts a recessive female fertility gene and CRISPR is exclusively active in differentiated germline cells. The daisy drive increases in frequency until most females are infertile due to inheriting a disrupted copy from both parents, at which point the population crashes. The finite number of daisy elements keeps suppression confined to the local population, which is a necessity for conservation applications. CRISPR, Clustered Regularly Interspaced Short Palindromic Repeats.

A potentially more general proposed solution involves building an inherently local drive system. In a “daisy drive” system, the drive components are linked in a daisy chain: D drives C drives B drives A (Fig 2) [30]. Each element in the chain functions as a form of genetic fuel: they are sequentially spent over generations of mendelian inheritance until the drive runs out and stops. Models suggest that releasing only a few organisms with daisy drive will eventually affect a fairly large population before halting, which means that the released animals themselves needn't cause much additional ecological damage [30]. Other daisy drive variants may offer further localization advantages [31]. Nor is daisy drive the only game in town; alternative approaches such as the “Trojan female technique” recently demonstrated in flies might also be adapted for local elimination [32].

These proposals are promising, but there is much to be done before any local drive system functions in rats, let alone nonmodel stoats and possums, and even more work to ensure that these systems will behave as intended. While New Zealand is a world leader in biosecurity, with a strong legislative and consultative process that restricts the environmental release of organisms perceived to pose undue risks, humanity has no experience engineering systems anticipated to evolve outside of our control, so we will need rigorous and transparent safety testing in the laboratory before considering field trials of even local drive systems.

## Open, community-guided eco-engineering research

Proponents of genetic solutions are correct to start the conversation now. Any technology capable of eliminating mammalian predators from New Zealand will by definition alter the shared environment. That means that all proposals and research should be open from the earliest stages, with scientists and supporters actively inviting dialogue with those who have reservations [33].

While acknowledging the daily toll that invasive predators inflict on our global biodiversity [34], we suggest that interested communities and conservationists help guide the development of local drive systems, always insisting on openness and community direction, rather than press forward with initiatives based on suboptimal standard gene drive systems [35]. The likely cost of impatience is simply too high. Even if daisy drive and equivalents don’t work well enough, interest from the Defense Advanced Research Projects Agency (DARPA) Safe Genes program and other funders makes it likely that a superior strategy will be invented soon.

Are New Zealand communities prepared to guide the development and oversee the testing of these systems? Are similar conversations happening internationally? We hope so, because this conversation should not be confined to scientists, regulators, politicians, or any single nation, no matter how strong its legislative frameworks, environmental risk management, and biosecurity networks [36]. If we have learned anything from the spread of invasive species, it is that ecosystems are connected in myriad ways and that a handful of organisms introduced in 1 country may have ramifications well beyond its own borders.

## Abbreviations

DARPA: Defense Advanced Research Projects Agency
PAM: protospacer-adjacent motif
CRISPR: Clustered Regularly Interspaced Short Palindromic Repeats
